# Seroprevalence and associated factors of HSV-2 infection among general population in Shandong Province, China

**DOI:** 10.1186/s12879-019-3995-2

**Published:** 2019-05-06

**Authors:** Pengcheng Huai, Furong Li, Zhen Li, Lele Sun, Xi’an Fu, Qing Pan, Gongqi Yu, Zemin Chai, Tongsheng Chu, Zihao Mi, Fangfang Bao, Honglei Wang, Bingni Zhou, Chuan Wang, Yonghu Sun, Guiye Niu, Yuan Zhang, Fanghui Fu, Xiaoqiao Lang, Xiaoling Wang, Hui Zhao, Daina Liu, Hong Liu, Dianchang Liu, Jian Liu, Aiqiang Xu, Furen Zhang

**Affiliations:** 1 Shandong Provincial Hospital for Skin Disease, Shandong University, Jinan, China; 2 Shandong Provincial Institute of Dermatology and Venereology, Shandong Academy of Medical Sciences, 27397 Jingshi Road, Jinan, 250022 China; 3 Department of Epidemiology, School of Public Health, Shandong University, Jinan, China; 4 Shandong Provincial Key Laboratory for Dermatovenereology, Jinan, China; 5 Shandong Center for Disease Control and Prevention, Jinan, China

**Keywords:** HSV-2 (*herpes simplex virus type-2*), Seroprevalence, Associated factors, China

## Abstract

**Background:**

Herpes simplex virus type-2 (HSV-2) infection is the main cause of genital ulcer disease and increases the risk of HIV acquisition. Little information is available regards the epidemiological characteristics of HSV-2 among general population in China. The aim of this study was to explore seroprevalence and associated factors of HSV-2 and provide information for design of HSV-2 control strategy in Shandong, China.

**Methods:**

In this cross-sectional study, a total of 8074 persons, 18–49 years of age, were selected using multi-stage probability sampling to represent the general population of Shandong in 2016. Demographic data were collected through face-to-face interviews. Other variables were obtained by self-administered questionnaire surveys. Blood was collected for HSV-2 IgG detection with ELISA.

**Results:**

A total of 7256 sexually-active participants were included in the analysis. The weighted seroprevalence of HSV-2 infection was 4.2% (95% confidence interval [CI], 3.2–5.3) in females, which was significant higher than that in males (2.7%; 95% CI, 1.1–4.2) (*P* = 0.04). The seroprevalence of HSV-2 was higher in individuals from eastern region (6.4%; 95% CI, 5.9–6.9) and urban areas (4.3%; 95% CI, 2.6–6.0) of Shandong than those from other regions (*P* < 0.01). Associated factors for HSV-2 infection among men were being urban residents (adjusted odds ratio [AOR], 2.36; 95% CI, 1.14–4.88), having two or more sex partners in the past year (AOR, 3.22; 95% CI, 1.90–5.43) and having commercial sex (AOR, 1.51; 95% CI, 1.00–2.26). Among females, being divorced or widowed (AOR, 1.79; 95% CI, 1.08–2.97), having a tattoo (AOR, 2.89; 95% CI, 1.07–7.84), and being dissatisfied with the sex activity quality (AOR, 2.12; 95% CI, 1.24–3.63) was associated with HSV-2 infection.

**Conclusions:**

This study showed a relatively low burden of HSV-2 in Shandong province, China compared with the seroprevalence reported in many other provinces and countries. HSV-2 control programs in Shandong should focus on eastern, urban and female residents, and pay more attention to individuals with identified associated factors.

## Background

Herpes simplex virus type-2 (HSV-2) infection is the main cause of genital ulcer disease worldwide [1]. In 2012, the estimated prevalence of HSV-2 among individuals 15–49 years of age was 11.3%, corresponding to 417 million infected cases in the word [2]. Approximately 88% of HSV-2 infected patients do not know they have the disease, but the asymptomatic nature of genital herpes facilitates the spread of the infection in the general population [3, 4]. The infection can be transmitted to the fetus during pregnancy, which is an important cause of neonatal herpes, death, and long-term disability [5]. In addition, HSV-2 increases the risk of HIV acquisition by 1.7 fold in men and 2.1 fold in women, and the risk is even higher in newly HSV-2 infected individuals [2, 6].

Syphilis and HIV have increased significantly in China from 2004 to 2013, with annual percentage changes of 16.3% for both diseases [7]. However, little information is available regards the prevalence or incidence of HSV-2 among the general population in China. A population-based survey provided an estimate of seroprevalence and associated factors, which facilitated the design of specific HSV-2 control program [8]. To our knowledge, only two studies estimated seroprevalence of HSV-2 infection among general population in China. The first survey was conducted in Zhejiang Province in 2006; but the participants did not include an urban population [9]. Another study conducted in Shanghai in 2011, which reported the seroprevalence of HSV-2 among 600 female residents; however, no males were included in this study [10]. Thus, it is essential to conduct a population-based study with a large sample size including males and females to determine the urban and rural seroprevalence of HSV-2 among the general population in China.

Since the implementation of the Chinese universal two-child policy in 2015, Chinese couples have had the freedom to have a second child [11]. More HSV-2 infected asymptomatic females who want to have their second baby may suffer from adverse complications. Therefore, targeted propaganda and education or other specific control interventions are needed to prevent HSV-2 infections and adverse outcomes among hish risk individuals before they got pregnant.

This study was conducted to estimate the seroprevalence and associated factors of HSV-2 and provide information for design of HSV-2 control strategy in Shandong Province.

## Methods

### Study design and participants

This population-based, cross-sectional study was conducted between May and August 2016. Details of the study design have been described previously [12]. A multi-stage complex sampling method was used to select participants who could represent the general population 18–49 years of age in Shandong Province, China. Shandong is a peninsular province located in eastern China. The population of Shandong was 98,470,000 in 2015, accounting for approximately 7% of the whole population in China. Shandong was divided into east, northwest, south, and middle based on geographic region; 12 urban districts or counties (primary sampling units [PSUs]) were selected randomly from the four strata. The selection of rural townships or urban street districts (subunits) from the PSUs and selection of rural villages or urban communities from subunits used the probability proportionate to size sampling (PPS) method. PPS method is often uesd in muti-stage complex sampling and the probability of selection for a unit is directly proportional to its size. While in proportional allocation, the probability of selection for a unit is the same for all strata. Rural villages and urban communities were selected at a proportion of 1:1. A total of 184 villages and 183 communities were included. Systematic sampling based on age and gender distribution of Shandong was conducted to select individuals from residents 18–49 years of age who had lived in the current residence for at least the past 6 months. Thus, 22 individuals were extracted from each village or community based on the sample size and number of selected villages or communities.

Inclusion criteria were as follows: 18–49 years of age by 31 September 2015; continuous residence at the study site for at least the past 6 months. Exclusion criteria were as follows: individuals who had not had their sexual debut; and could not provide correct information due to mental illness or intoxication.

The study was approved by the Ethics Committee of Shandong Provincial Institute of Dermatology and Venereology (approval number: 2016–04). Individuals who were willing to provide blood and answer pertinent questions were recruited in this study. Before each interview, oral informed consent was obtained from the participant.

### Procedures

Three training days were provided to the fieldwork staff in each of the PSUs. Each field team included one laboratory technician for blood collection, two questionnaire interviewers, and one financial staff for distribution of the interview allowance. In addition, one trained supervisor from the research group was also designated to each field team for informed consent and quality control.

We performed the investigation in a village/community clinic or meeting room of the village/community committee. Demographic information, including sex, age, marriage (unmarried, married, divorced, or widowed), and rural or urban residence, was collected by face-to-face interview. Other information was completed by the participants themselves. To increase the response rate, investigations were usually performed early in the morning or late in the evening because many individuals work during the day. The supervisor in each field team checked the questionnaires for missing value and logical error and wrote a unique alphanumeric code on each questionnaire after the interview.

Five milliliters of venous blood was collected from each participant by a skilled laboratory technician. Blood was put in a styrofoam cooler and transported to the local laboratory within 3 h. Serum was separated from blood and frozen at − 20 °C upon receipt of the specimen. Serum was put in the styrofoam cooler and transported to the laboratory at Shandong Provincial Institute of Dermatology and Venereology within 2 weeks.

We tested serum for HSV-2 using an enzyme-linked immune sorbent assay (ELISA) for IgG antibodies. According to the manufacturer instructions, the sensitivity, specificity, false positive rate and false negative rate of this test were 97.3, 98.1, 1.9 and 2.7% respectively. Result was valid when optical density (OD) value of positive control ≥1.00, OD value of negative control ≤0.05, and OD value of cut-off control ≥0.15 for each test. OD value of sample more than that of cut-off control was deemed positive. Many other population-based studies on HSV-2 seroprevalence used the same method [3, 13]. Individuals with positive tests results were referred to a local STI clinic, genitourinary clinic, or general hospital for treatment.

### Operational definition

The self-designed structured questionnaires included variables on tattooing, smoking (smokers defined as those who had smoked 100 or more cigarettes during lifetime), times of drunk in the past 12 months (0, 1–3, 4–6, and > 6), domestic violence, history of sexually transmitted infections (STIs) in the past 5 years, quality of sex activity (subjective satisfied or not satisfied), sexual behaviors, such as age at sexual debut (≤20 years and > 20 years), extramarital sex, commercial sex and number of sex partners (1 and ≥ 2).

### Statistical analysis

All survey data were double-entered into Epidata 3.1 (EpiData association, Odense, Denmark). We analyzed data using *surveyfreq* and *surveylogistic* methods (SAS 9.3; SAS Institute Inc., Cary, NC, USA). The seroprevalence of HSV-2 was estimated with application of selection probability, non-response, and post-stratification weights. The 95% confidence interval (CI) for the seroprevalence of STIs was calculated based on total population weights and Taylor series linearization. Independent associations between potential associated factors and HSV-2 infection were explored with logistic regression and reported as the crude odds ratio (COR) in bivariate analysis. For multivariate logistic regression model analysis, associated factors with a *P* value < 0.10 in bivariate analysis, as well as age, were included in the model [14]. Adjusted ORs (AOR) and 95% CIs were presented. In addition, the variance inflation factor, condition index, and variance proportions were calculated to examine potential collinearity. All statistical tests were two-sided, and variables with *P*-value < 0.05 were considered statistically significant.

## Results

**Fig. 1:**
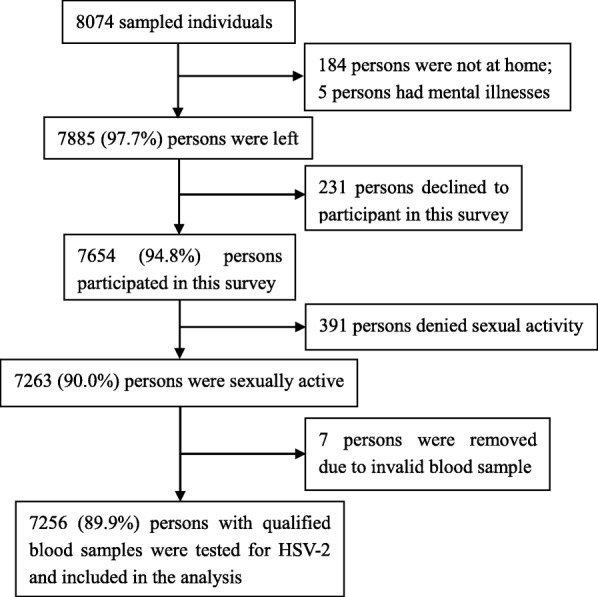
Flow chart of participant selection in Shandong province, China in May to August, 2016. Abbreviations: HSV-2 herpes simplex virus type-2

Of the 8074 individuals originally sampled, 189 were removed because of mental illness or not available, 231 individuals declined to take part in the study, and 391 had not had their sexual debut. Thus, 7263 (90.0%) individuals completed the survey and provided blood samples. Seven invalid blood samples were excluded, leaving 7256 (89.9%) qualified blood samples with test results included in the analysis (Fig. 1).

### Seroprevalence of HSV-2

The weighted age, gender and region-specific seroprevalence of HSV-2 is shown in Table 1. Of the 7256 blood samples tested, 254 participants (88 males and 166 females) tested positive for HSV-2. The estimated seroprevalence of HSV-2 in the general population 18–49 years of age in 2016 was 3.4% (95% CI, 2.2–4.7), 2.7% (95% CI, 1.1–4.2) for males, and 4.2% (95% CI, 3.2–5.3) for females, corresponding to ~ 1,735,000 total cases (95% CI, 1,058,000–2,411,000) who had been infected with HSV-2 in Shandong. The HSV-2 seroprevalence for females was significantly higher than males (*P* = 0.04). The highest seroprevalence of HSV-2 for males was in males 35–44 years of age (3.6%; 95% CI, 2.5–4.8). For females, the highest seroprevalence of HSV-2 was in those 30–34 years of age (6.9%; 95% CI, 5.0–8.8). The seroprevalence of HSV-2 was higher in males (6.2%; 95% CI, 5.2–7.3) and females (6.5%; 95% CI, 5.1–8.0) from Eastern region of Shandong (6.4%; 95% CI, 5.9–6.9) than those from other regions (*P* < 0.01 for both genders). The seroprevalence of HSV-2 for urban residents was 4.3% (95% CI, 2.6–6.0), which was significantly higher than rural residents 2.4% (95% CI, 1.9–2.8, *P* < 0.01).

**Table 1:** The weighted seroprevalence of HSV-2 in participants 18–49 years of age by gender, age group and region in Shandong province, China in May to August, 2016

	Male	Female	Denominator^a^ (unweighted, weighted^b^)	
	[n, %(95% CI)]	[n, %(95% CI)]	Male	Female
Age (years)				
18–24	6, 2.4% (0.0–5.9)	9, 3.2% (0.9–5.4)	253, 852	195, 828
25–29	12, 1.4% (0.5–2.3)	31, 3.1% (2.2–4.0)	786, 500	914, 499
30–34	10, 2.5% (0.0–5.2)	33, 6.9% (5.0–8.8)	503, 471	559, 465
35–39	19, 3.6% (1.4–5.8)	29, 4.6% (2.9–6.3)	566, 575	591, 561
40–44	27, 3.6% (1.7–5.6)	36, 4.7% (2.1–7.4)	721, 675	742, 668
45–49	14, 2.3% (0.0–4.7)	28, 3.5% (2.0–5.1)	697, 578	729, 584
Region				
Northwest	6, 1.4% (1.0–1.8)	10, 2.6% (0.7–4.6)	340, 393	349, 358
Middle	14, 1.4% (1.2–1.6)	33, 2.9% (1.9–4.0)	902, 949	916, 861
South	28, 1.8% (1.3–2.3)	63, 3.9% (2.5–5.4)	1505, 1469	1582, 1493
East	40, 6.2% (5.2–7.3)	60, 6.5% (5.1–8.0)	779, 840	883, 893
Total	88, 2.7% (1.1–4.2)	166, 4.2% (3.2–5.3)	3526, 3651	3730, 3605

*Abbreviations: CI* confidence interval

^a^Denominator was participants with sex experience and a blood test result

^b^Selection probability weighting, non-response weighting, and post-stratification weighting were applied to calculate the weighted denominator

### Associated factors for HSV-2 infection

With respect to males, bivariate analyses showed that the HSV-2 seroprevalence was higher for the following: urban residents; those who smoking; those who had imposed domestic violence on their spouses; those with a transfusion history; those who had engaged in extramarital or commercial sex; and those who had > 2 sex partners in the past year (Table 2).

**Table 2:** Associated factors for HSV-2 among men 18–49 years of age in Shandong province, China in May to August, 2016

	% (95% CI)	Crude OR (95% CI)	Adjusted OR (95% CI)^a^	Denominator^b^ unweighted, weighted
All ages	2.7% (1.1–4.3)	–	–	3526, 3651
Age (years)	–	*p* = 0.41	*p* = 0.14	–
18–24	2.4% (0.0–5.9)	1.00	1.00	253, 852
25–29	1.4% (0.5–2.3)	0.59 (0.22–1.57)	0.74 (0.26–2.10)	786, 500
30–34	2.5% (0.0–5.2)	1.06 (0.53–2.12)	1.42 (0.70–2.89)	503, 471
35–39	3.6% (1.4–5.8)	1.56 (0.27–8.91)	2.17 (0.33–14.11)	566, 575
40–44	3.6% (1.7–5.6)	1.56 (0.50–4.83)	2.19 (0.66–7.28)	721, 675
45–49	2.3% (0.0–4.7)	0.98 (0.46–2.08)	1.39 (0.63–3.05)	697, 578
Marital status		*p* = 0.92	–	
Unmarried/married	2.7% (1.0–4.3)	1.00	–	3473, 3602
Divorced/widowed	2.4% (0.0–6.7)	0.91 (0.16–5.13)	–	53, 49
Location of residence	–	*p* = 0.02	*p* = 0.02	–
Rural	1.5% (0.7–2.3)	1.00	1	1799, 2082
Urban	3.6% (1.3–5.8)	2.44 (1.12–5.26)	2.36 (1.14–4.88)	1727, 1569
Smoking		*p* = 0.08	*p* = 0.14	–
No	2.2% (0.5–4.0)	1.00	1	1761, 1796
Yes	3.1% (0.0–5.2)	1.39 (0.96–2.04)	1.34 (0.91–1.99)	1765, 1855
Times of drunk in the past year		*p* = 0.68	–	–
0	3.1% (0.0–7.0)	1.00	–	1042, 1058
1–3	2.0% (1.3–2.7)	0.62 (0.18–2.08)	–	1049, 1073
4–6	3.2% (1.5–4.9)	1.03 (0.36–2.94)	–	289, 329
> 6	2.0% (0.0–3.9)	0.62 (0.11–3.42)	–	317, 339
Domestic violence to wife^c^	–	*p* < 0.01	–	–
No	2.4% (1.2–3.7)	1.00	–	2928, 2845
Yes	4.9% (1.0–8.8)	2.06 (1.35–3.14)	–	318, 275
Transfusion history	–	*p* = 0.05	*p* = 0.15	–
No	2.6% (1.0–4.1)	1.00	1.00	3364, 3481
Yes	5.1% (0.6–9.5)	2.03 (1.01–4.09)	1.61 (0.85–3.07)	162, 170
Quality of sex activity		*p* = 0.59	–	–
Satisfied	2.7%(0.9–4.6)	1.00	–	3238, 3386
Dissatisfied	2.0%(0.6–3.5)	0.73 (0.24–2.27)	–	288, 266
Extramarital sex^c^	–	*p* < 0.01	–	–
No	2.4% (1.0–3.8)	1.00	–	2926, 2794
Yes	4.6% (2.6–6.5)	1.93 (1.53–2.45)	–	320, 326
Number of sex partners in the past year	–	*p* < 0.01	*p* < 0.01	–
< 2	2.3% (0.9–3.6)	1.00	1.00	3302, 3349
≥ 2	7.1% (1.5–12.7)	3.28 (2.05–5.27)	3.22 (1.90–5.43)	224, 302
Commercial sex	–	*p* < 0.01	*p* = 0.05	–
No	2.5% (1.0–4.0)	1.00	1.00	3382, 3463
Yes	6.3% (0.8–11.9)	2.66 (1.65–4.29)	1.51 (1.00–2.26)	144, 188

*CI* confidence interval, *OR* odds ratio

^a^Adjusted for age, location of residence, smoking, transfusion history, number of sex partners in the past year, and commercial sex

^b^Denominator was participants with sex experience and a blood test result

^c^ Those variables were not included into multivariate analyses because of missing data for unmarried participants

With respect to females, bivariate analyses indicated that increased HSV-2 seroprevalence was associated with being divorced or widowed, having a tattoo, being dissatisfied with the quality of their sex activity, and having > 2 lifetime sex partners (Table 3).

**Table 3:** Associated factors for HSV-2 among women 18–49 years of age in Shandong province, China in May to August, 2016

	% (95% CI)	Crude OR (95% CI)	Adjusted OR (95% CI)^a^	Denominator^b^ unweighted, weighted
All ages	4.2% (3.2–5.3)	–	–	3730, 3605
Age (years)	–	*p* = 0.57	*p* = 0.59	–
18–24	3.2% (0.9–5.4)	1.00	1.00	195, 828
25–29	3.1% (2.2–4.0)	0.98 (0.51–1.88)	1.04 (0.51–2.14)	914, 499
30–34	6.9% (5.0–8.8)	2.27 (0.99–5.22)	2.46 (0.94–6.44)	559, 465
35–39	4.6% (2.9–6.3)	1.48 (0.74–2.95)	1.61 (0.68–3.80)	591, 561
40–44	4.7% (2.1–7.4)	1.52 (0.59–3.94)	1.62 (0.55–4.79)	742, 668
45–49	3.5% (2.0–5.1)	1.13 (0.44–2.89)	1.18 (0.38–3.63)	729, 584
Marital status	–	*p* = 0.01	*p* = 0.02	–
Unmarried/married	4.2% (3.1–5.2)	1.00	1.00	3678, 3564
Divorced/widowed	8.8% (4.2–13.3)	2.21 (1.21–4.04)	1.79 (1.08–2.97)	52, 41
Location of residence	–	*p* = 0.10	–	–
Rural	3.2% (2.0–4.5)	1.00	–	1898, 1553
Urban	5.0% (3.5–6.4)	1.56 (0.92–2.64)	–	1832, 2052
Smoking	–	*p* = 0.47	–	–
No	4.2% (3.2–5.3)	1.00	–	3660, 3551
Yes	2.4% (0.0–6.2)	0.56 (0.12–2.70)	–	70, 54
Domestic violence from husband	–	*p* = 0.63	–	
No	4.1% (2.9–5.3)	1.00	–	3406, 3141
Yes	4.7% (2.6–6.8)	1.15 (0.67–1.97)	–	195, 176
Transfusion history		*p* = 0.12	–	–
No	4.3% (3.3–5.4)	1.00	–	3533, 3398
Yes	2.7% (0.7–4.7)	0.61 (0.33–1.14)	–	197, 207
Having a tattoo	–	*p* = 0.04	*p* = 0.04	–
No	4.1% (3.1–5.2)	1.00	1.00	3684, 3569
Yes	11.9% (0.9–22.9)	3.14 (1.07–9.18)	2.89 (1.07–7.84)	46, 36
Quality of sex activity	–	*p* < 0.01	*p* < 0.01	–
Satisfied	3.9% (2.8–5.0)	1.00	1.00	3510, 3389
Dissatisfied	8.5% (4.9–12.1)	2.26 (1.35–3.79)	2.12 (1.24–3.63)	220, 216
Extramarital sex		*p* = 0.68	–	–
No	4.1% (2.8–5.4)	1.00	–	3471, 3197
Yes	5.1% (1.0–9.1)	1.24 (0.44–3.50)	–	130, 121
Number of sex partners	–	*p* = 0.07	*p* = 0.15	–
< 2	4.1% (3.1–5.0)	1.00	1.00	3617, 3461
≥ 2	8.0% (1.7–14.2)	2.05 (0.95–4.44)	2.17 (0.76–6.23)	113, 144
Commercial sex	–	*p* = 0.86	–	
No	4.2% (3.2–5.2)	1.00	–	3688, 3572
Yes	4.6% (0.0–9.8)	1.09 (0.41–2.91)	–	42, 33

“Times of drunk in the past year” was not presented in the table because 91% of participants did not drink in the past year

*CI* confidence interval, *OR* odds ratio

^a^Adjusted for age, marital status, having a tattoo, quality of sex activity, and number of sex partners

^b^Denominator was participants with sex experience and a blood test result

Based on multivariate analyses, factors remaining significantly associated with HSV-2 infection for males were urban residence (AOR, 2.36; 95% CI, 1.14–4.88), having > 2 lifetime sex partners (AOR, 3.22; 95% CI, 1.90–5.43) and having commercial sex (AOR, 1.51; 95% CI, 1.00–2.26; Table 2). For females, being divorced or widowed (AOR, 1.79; 95% CI, 1.08–2.97), having a tattoo (AOR, 2.89; 95% CI, 1.07–7.84), and being dissatisfied with the quality of one’s sex activity (AOR, 2.12; 95% CI, 1.24–3.63) remained significantly associated with HSV-2 infections (Table 3).

Based on the collinearity diagnosis, the maximal variance inflation factor was 1.15 and the maximal condition index was 1.48 for the male regression model, while the maximal variance inflation factor was 1.02 and the maximal condition index was 1.17 for the female regression model, which indicated no multi-collinearity based on the multivariate logistic analysis.

## Discussion

To our knowledge, this is the first study with a large sample size to determine the seroprevalence and associated factors for HSV-2 among general populations in Shandong Province, China. Overall, the weighted seroprevalence of HSV-2 for females 18–49 years of age was significantly higher than that for males in this study. HSV-2 infection was more prevalent among people from urban and eastern region of Shandong. In addition, associated factors of HSV-2 infection were also identified for both genders. These findings were valuable for design HSV-2 prevention and control intervention in Shandong province.

Compared with the two previous population-based HSV-2 seroprevalence surveys conducted in Zhengjiang and Shanghai, we found a spatial heterogeneity of HSV-2 distribution in China [9, 10]. The unweighted seroprevalence of HSV-2 was 13.5% (95% CI, 11.8–15.2) among the general population 15–49 years of age in Zhejiang in 2006 [9]. In our study, seroprevalence of HSV-2 among residents 18–49 years of age (3.4, 95% CI, 2.2–4.7) was about one quarter of those in Zhejiang. For female residents 18–65 years of age in Shanghai, the unweighted seroprevalence of HSV-2 was 15.3% (95% CI, 12.4–18.2) in 2011, which was about 2.6 times higher than those in females 18–49 years of age in our study [10]. Both Zhejiang and Shanghai were located in southeastern coastal China. The high HSV-2 seroprevalence in the two regions may be due to large number of migrants. It is possible that migrants who had high-risk behaviors may spread STIs to the general population when moving from one place to another [15]. Therefore, HSV-2 control programs in China should be based on geographical regions.

We also compared the results of this study with other HSV-2 seroprevalence studies in American or European countries. HSV-2 infection is prevalent in 12.1% (95% CI, 9.7–15.0) of adults 14–49 years of age in the USA in 2015–2016, which is > 3 times what we observed [16]. Another national population-based survey conducted in Peru showed that the HSV-2 seroprevalence was 10.0% (95% CI, 9.6–10.4) and 17.4% (95% CI, 16.9–17.9) for sexually experienced males and females [13]. Balaeva T et al. performed a study of 1243 adults aged 18–39 years in Russia, which reported a HSV-2 seroprevalence of 18.8% (95% CI, 16.8–21.1) [3]. A fourth study conducted by Woestenberg PJ in the Netherlands in 2006–2007 showed that the HSV-2 seroprevalence of general Dutch population was 6.0% (95% CI, 4.8–7.2) [17]. Compared the results of these surveys, we found a approximate 3 to 16% lower HSV-2 seroprevalence in our study. However, despite lower seroprevalence, the number of infected cases was substantial due to large population size of Shandong.

Most studies, including the current study, demonstrated that having multiple sex partners is associated with HSV-2 infection [3, 18–20]. The findings of the current study indicated that risk for HSV-2 infection among males with > 2 sex partners was 3 times as high as those with < 2 sex partners in the past year. The number of lifetime sex partners was also associated with HSV-2 infection for females, although the strength of the association was weak. Extramarital or commercial sexual behaviors increased the number of sex partners for males and increased the risk of HSV-2 infection in the current study. Thus, males who have multiple sex partners were suggested to be tested for HSV-2 infection regularly.

Having a tattoo has been identified to be associated with HIV, hepatitis C virus (HCV), and HSV-2 infections [18, 21, 22]. The current study showed that female residents who had a tattoo had a risk for HSV-2 infection that was ~ 3 times higher than those who did not have a tattoo. Tattooing is a way for people to express liberal attitudes towards life and sexuality [23]. Having a tattoo has been identified to be associated with being sexually active, early sexual initiation and a higher number of lifetime sexual partners, which increase the risk of STIs infection [24, 25]. We estimated that nearly 1% of female residents 18–49 years of age had a tattoo. Thus, this small percentage of high-risk individuals should be given greater emphasis when designing a HSV-2 control strategy.

Our study suggested that females who were divorced or widowed had a higher probability of HSV-2 infection. Several other surveys have also shown the same relationship between marital status and HSV-2 infection, particularly for females [3, 26, 27]. A study from Tanzania showed that females who were divorced or widowed may engage with multiple sex partners due to relative personal freedom and in an attempt to establish new relationships [27]. Another study from Kenya inferred that some of such females may experience significant pressure due to childrearing and few skills to earn money, thus some of them may resort to transactional sex [26]. However, the reason why females who were divorced or widowed had a relatively higher seroprevalence of HSV-2 infection in Shandong still need further research.

Data collected in this study indicated that females with a poor quality of sex activity area high-risk population for infection with HSV-2. Researchers from Iran have reported that a lower level of sexual satisfaction can decrease the quality of marital life and increase the divorce rate [28]. Another study from Kenya showed an association between poor sexual satisfaction and extramarital partnerships [29]. Indeed, we have discussed that individuals who are divorced or have extramarital partnerships usually have multiple sex partners, which increases the risk to be infected with HSV-2. On the other hand, those women may be transmitted by their husbands who may engage in extramarital sexual behaviors due to dissatisfaction in couple’s sex activity. We advised that females with a poor quality of sex activity to tested HSV-2 infection regularly.

There were several limitations in our study. First, individuals who migrated to their current residence and lived there for > 6 months were hard to completely registered and sampled. Thus, the seroprevalence of HSV-2 may be underestimated because migrants were identified as high risk population of STIs infection [30]. Second, seroprevalence of HSV-2 among individuals less than 18 year of age and older than 49 years of age was not estimated in this study. Further investigation exploring seroprevalence of HSV-2 as well as other STIs among these people is recommended. Third, the sample size was relatively small for a few of sub-groups, such as 18–24 year old group, which resulted in large 95% CI and added some uncertainty to the estimates.

## Conclusions

This study showed a relatively low burden of HSV-2 (seroprevalence: 3.4%) in Shandong province, China compared with the seroprevalence reported in many other provinces and countries, including European countries and the Americas. The design and delivery of HSV-2 prevention and control intervention in Shandong should focus on eastern, urban and female residents, and pay more attention to individuals with identified associated factors.

## Abbreviations

CI: Confidence interval
ELISA: Enzyme-linked immune sorbent assay
HCV: Hepatitis C virus
HSV-2: Herpes simplex virus type-2
OD: Optical density
OR: Odds ratio
PPS: Probability proportionate to size sampling
PSU: Primary sampling unit
STIs: Sexually transmitted infections
VIF: Variance inflation factor
